# Emotional learning in older adults with and without Alzheimer's Disease

**DOI:** 10.1002/alz70857_104127

**Published:** 2025-12-25

**Authors:** Sophia I Vecchi, Martha Escobar

**Affiliations:** ^1^ Oakland University, Rochester Hills, MI, USA

## Abstract

**Background:**

Despite their explicit memory difficulties, individuals with Alzheimer's Disease (AD) exhibit implicit memory (Moran, 2023). We assessed whether evaluative conditioning, in which irrelevant stimuli paired with stimuli eliciting positive or negative emotions, evoke an emotional response (a type of implicit memory), is preserved in older adults with and without AD.

**Method:**

Participants were older adults (aged 65+) diagnosed with AD (Group AD), type 2 diabetes (Group DB), or without neurodegenerative disease or diabetes diagnoses (Group ND). Group DB was included because diabetes increases the risk of developing AD. All participants had a minimum score of 15 (mild memory impairment) in the Self‐Administered Gerocognitive Exam (SAGE). Participants viewed a set of fictitious books on a computer screen. For each book, they first saw the cover (a colored pattern with foreign characters), and then saw and heard a brief summary of the story, which could be pleasant (e.g., a lost puppy finds its way home) or unpleasant (e.g., a lost puppy is never found). Unbeknownst to participants, one of the features of the book covers (e.g., red letters) was associated with pleasant stories, and a different feature (e.g., blue letters) with unpleasant stories. Then, participants were presented with a book cover, and asked to rate how pleasant they expected the story to be. They were also asked to recall as many of the stories as they could.

**Result:**

It is expected, based on previous research, Group AD will exhibit little recall of the stories, and will be unlikely to explicitly link a characteristic of the book cover with the type of story it contains, but should not differ from Group ND on predicting whether it will be a pleasant or unpleasant story (Maturana, 2023). Group DB's performance in the explicit test should be similar or lower than in Group ND, but should show no deficits in the implicit test.

**Conclusion:**

This study is expected to replicate the dissociation between explicit and implicit memory. As a new contribution, it will determine whether evaluative conditioning is a valid tool to assess emotional learning in individuals with dementia.

